# Clinical Phenotype and Genetic Analysis of a Family with Hereditary Antithrombin Deficiency Caused by SERPINC1 Gene Mutation

**DOI:** 10.1055/a-2558-8193

**Published:** 2025-04-09

**Authors:** Yating Zhao, Longting Du, Shaobin Lin, Lu Bai, Yao Chen, Manman Ye, Shihong Zhang, Chang Su, Xiaohe Zheng

**Affiliations:** 1 Department of Laboratory Medicine, The First Affiliated Hospital, Sun Yat-Sen University, Guangzhou, China; 2 Department of Obstetrics and Gynecology, Prenatal Diagnosis Center, The First Affiliated Hospital, Sun Yat-sen University, Guangdong, China; 3 Department of Hematology, The First Affiliated Hospital, Sun Yat-Sen University, Guangzhou, China

**Keywords:** antithrombin, inherited AT deficiency, SERPINC1 gene, PROC gene, mutation

## Abstract

**Background:**

Inherited deficiency of the antithrombin (hereditary antithrombin deficiency, AT deficiency, OMIM #613118) is a relatively rare (1:2,000–3,000) autosomal-dominant disorder with high risk of venous thromboembolism. The molecular basis of this condition has not yet fully understood, highlighting the need for further research to elucidate the underlying pathological mechanisms.

**Objective:**

This study aimed to investigate coagulation parameters and genetic phenotypes in a proband with hereditary antithrombin deficiency and her family members. Additionally, the investigation sought to provide preliminary insights for the molecular pathogenesis of this condition.

**Methods:**

Blood coagulation parameters, including plasma antithrombin activity (AT:A), antithrombin antigen (AT:Ag), protein C activity (PC:A), and protein S activity (PS:A) were measured in the peripheral blood of each family member by a Stago instrument. Peripheral blood was also extracted and sequenced to identify possible genetic mutation sites. The functional impact of variants on protein was subsequently analyzed by bioinformatics software.

**Results:**

The proband, her mother, and brother all exhibited decreased activity and antigen of AT but normal PC and PS activity. The proband's father had normal activity and antigen levels of AT, PC, and PS. Sequencing revealed the proband's mother inherited the SERPINC1:c.661T > C,p.(Trp221Arg) heterozygous variant and her father harbored PROC:c.572_574del,p.(Lys193del) heterozygous variant while the proband as well as her brother carried both. Conservation analysis revealed that Trp221 is highly conserved across homologous species. Bioinformatics tools consistently classify the p.Trp221Arg mutation as “pathogenic” or “deleterious.” Protein modeling indicated that the p.Trp221Arg variant does not alter the protein structure but may modify glycosylation sites to affect its function.

**Conclusion:**

The proband and family members exhibited varying degrees of decreased levels of AT and thrombosis, which were closely associated with inheritance of SERPINC1:c.661T > C,p.(Trp221Arg).

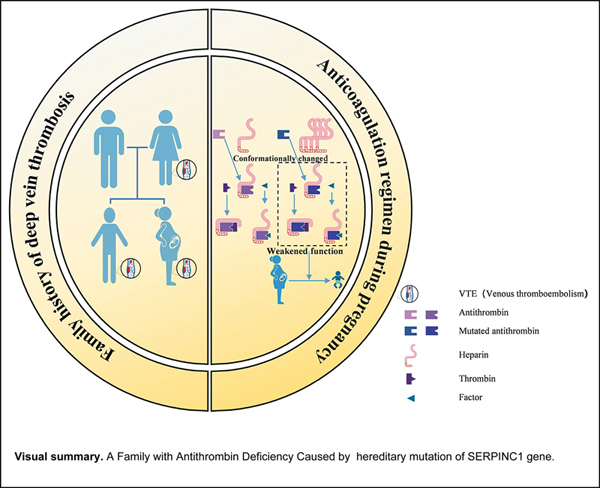

## Introduction

Antithrombin (AT) is a natural anticoagulant that is produced by hepatocytes and endothelial cells within the body. It is a small, single-chain glycoprotein that is composed of a total of 432 amino acids. 1 The primary function of AT is to specifically bind to the serine protease active sites of thrombin and various other coagulation factors, which inhibits their activity and results in an anticoagulant effect within the body. 2 AT, as the most essential endogenous anticoagulant, also inhibits all other procoagulant serine proteases in addition to inhibiting thrombin. 3 Heparin, acting as a cofactor, intensifies the inhibitory effect of AT on its target proteases, meaning that even a slight deficiency in AT can substantially raise the risk of thrombosis. 4 The gene that encodes for AT is known as SERPINC1 and is located on chromosome 1q23-25. This gene consists of a total of six introns and seven exons. 5

Hereditary AT deficiency is a congenital coagulation disorder, which significantly increases the risk of venous thrombosis. Among patients diagnosed with venous thromboembolism (VTE), the prevalence is estimated to reach approximately 1%. 6 Most of AT-deficient patients carry heterozygous SERPINC1 variants. 7 Studies conducted in an experimental mouse model have demonstrated that a homozygous null variant in the SERPINC1 gene leads to embryonic lethality, attributed to bleeding complications and severe thrombosis. 8 Over 300 genetic variants responsible for AT deficiency have been discovered for the SERPINC1 gene. 6 Variants of SERPINC1 gene lead to abnormal levels of AT in the peripheral blood. 9

Hereditary AT deficiency usually follows an autosomal dominant inheritance pattern, which is characterized by either abnormal AT function or concentration as observed in laboratory tests. There are two main types of hereditary AT deficiency. Type I is caused by impaired AT synthesis, which results in concurrent reductions in both AT activity and antigen levels. Type II is caused by structural abnormalities in AT, which results in reduced AT activity despite normal antigen levels. 10

This particular study examines a family that has been diagnosed with hereditary AT deficiency. It analyzes the clinical phenotypes and genotypes of all the members of the family. Furthermore, the study also explores the potential molecular pathogenesis through the use of bioinformatics predictions. This will help in gaining a better understanding of the disorder and may also help in the development of potential treatments or interventions for the family affected by this condition.

## Patients and Methods

**Fig. 1:**
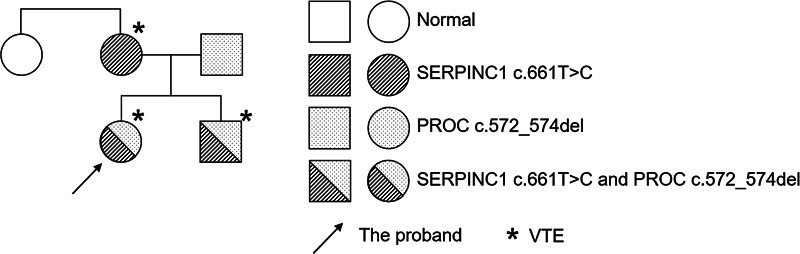
Family tree of the proband. *VTE, venous thromboembolism.

The proband was a 33-year-old female who presented for follow-up after more than 6 months of anticoagulant therapy following surgery for deep vein thrombosis (DVT) in the lower extremities. In May 2022, the patient experienced syncope suddenly and was admitted to the emergency department during the 8th week of early pregnancy. She complained of a sensation of swelling and pain in her left calf about a week ago, which was relieved by rest and did not receive any specific treatment. An outpatient Doppler ultrasound later confirmed a DVT in the left lower extremity, specifically within the calf muscle veins. On May 9, an inferior vena cava filter was placed, during which a DVT was also identified in the right lower extremity. On May 15, the patient underwent bilateral lower limb venography, mechanical thrombectomy, and catheter-directed thrombolysis. The patient's obstetric history was noted as gravida 1, para 0, with a previous history of one missed abortion. To find out the cause of thrombosis, the proband was screened for hereditary thrombophilia risk factors. Results revealed normal protein S (PS) and protein C (PC) activities, but a reduced AT activity of 49%. The study included the proband and three other family members across two generations. Both the proband's mother and younger brother also exhibited reduced AT activity, with normal PC and PS activities. The pedigree chart is shown in Fig. 1 .

## Sample Collection

Peripheral venous blood was drawn from each family member into three tubes. One tube collected 2.7 mL of blood mixed with 0.3 mL of sodium citrate anticoagulant (concentration: 109 mmol/L). The mixture was then centrifuged at 4°C, 1500 × g for 10 minutes. The upper layer of plasma was carefully collected for coagulation parameter testing. The second tube collected 2 mL of blood into an EDTA anticoagulant tube for genomic DNA extraction. The third tube collected 2 mL of blood into an EDTA anticoagulant tube for AT antigen.

## Plasma Coagulation Parameter Testing

Activated partial thromboplastin time (APTT), prothrombin time (PT), fibrinogen (FIB), thrombin time (TT), and protein S activity (PS) were assessed using the one-stage coagulation method. D-dimer (D-D) levels were determined by immunoturbidimetry, AT activity by chromogenic substrate assays, and protein C activity (PC) by clotting assays. All measurements were performed using a Stago coagulation analyzer (Stago, France) with corresponding reagent kits, strictly adhering to the manufacturers' instructions.

## Bioinformatics Analysis

The impact of gene variants on protein structure and function was predicted using bioinformatics tools such as Mutation Taster, PolyPhen-2, and LRT. The conservation of the mutated amino acids among six homologous species was analyzed using the ClustalX-2.1-win multiple sequence alignment software. The AT protein model template (PDB ID: P01008) was retrieved from existing protein databases, and the structures of wild-type and mutant AT proteins were modeled using Swiss-PdbViewer software. Comparative analysis was performed to assess the effects of the mutation on the spatial conformation and intermolecular interactions of the AT protein. Glycosylation analysis was performed using NetNGlyc 1.0.

## Result

### Coagulation Tests

Routine coagulation tests for the proband, including APTT, PT, fibrinogen (FIB), and TT, were all within normal limits. AT activity levels were 49% in the proband, 50% in her brother, and 42% in their mother, while their father was normal (104%). PC and PS activities were normal in all family members. The proband as well as her mother and younger brother all suffered from thrombosis before ( Table 1 ).

**Table 1:** The phenotype and thrombosis history of the proband and her parents and brothers

Family members	Age (years)	AT:A (%)	AT:A g (%)	PC:A (%) (clotting assay)	PC:A (%) (chromogenic assay)	PS:A (%)
Proband	33	49	51	103	107	89
Mother	56	42	42	138	133	74
Father	57	104	121	77	104	83
Brother	31	50	<31	107	110	84

Abbreviations: AT:A, antithrombin activity; AT:Ag, antithrombin antigen; PC:A, protein C activity; PS:A, protein S activity.

Note: Reference range: AT:A, 80–120%; AT:Ag, 80–120%; PC:A, 70–130%; PS:A; Male: 77–143%; Female: 55–123%.

### Results of SERPINC1 Gene Testing

All family members of the proband, except for the proband's father, carried the NM_000488.4(SERPINC1):c.661T > C,p.(Trp221Arg) heterozygous variant ( Table 2 ), which was co-segregated with AT deficiency in this family. The variant is absent from the existing population databases including gnomAD, 1000 Genomes, ESP 6500, and TOPMed Bravo databases. This variant has not been reported in the HGMD ( http://www.hgmd.org ), OMIM, or PubMed databases. The variant has a REVEL score of 0.88, supporting a deleterious effect on the gene or gene product.

**Table 2:** The genotype data of the proband and her parents and brothers

Family members	SERPINC1 c.661T > C (p.Trp221Arg)	PROC c.572_574del (p.Lys193del)
Proband	+	+
Mother	+	−
Father	−	+
Brother	+	+

The proband, her father, and her younger brother all harbored the NM_000312.4(PROC):c.572_574del,p.(Lys193del) heterozygous variant. This variant has a population frequency of 0.0672% in the gnomAD(all) database, within 188 heterozygous and 1 homozygous individuals. In the East Asian population, the frequency is 0.9473%, with 187 heterozygous and 1 homozygous individuals. The ClinVar database contains four records of this variant (Variation ID: 225448), classified as pathogenic (one) and of uncertain significance (three).

## Bioinformatics Analysis Results

### Conservation Analysis

**Fig. 2:**
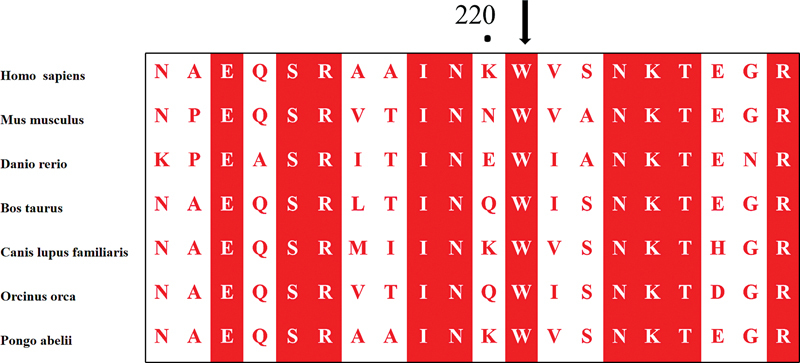
Clustal W alignment among the representative species at the sites of p.Trp221Arg.

All affected members carried the heterozygous missense variant c.661T > C,p.(Trp221Arg) in exon 4 of the SERPINC1 gene. A comparative analysis of homologous protein sequences from humans and other species (mouse, cattle, sheep, chimpanzee, rat, and zebrafish) revealed that tryptophan at position 221 is highly conserved ( Fig. 2 ).

### Simulation and Analysis of Protein Spatial Structure

**Fig. 3:**
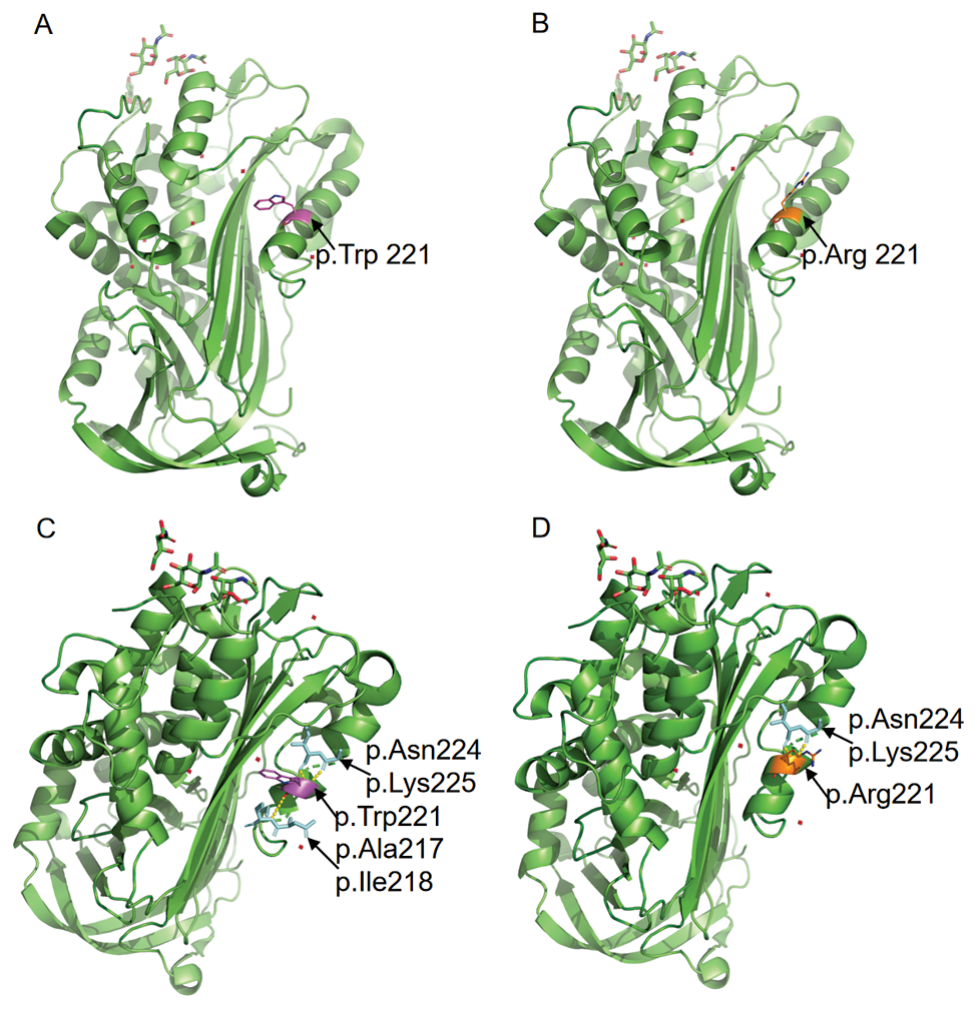
Modeling of the human antithrombin protein. ( **A** ) The structure of wild-type AT protein (p.TRP 221), ( **B** ) The structure of mutant AT protein (p.Arg 221), ( **C** ) The structure of hydrogen bonds in wild-type AT protein, ( **D** ) The structure of hydrogen bonds in mutant AT protein.

**Fig. 4:**
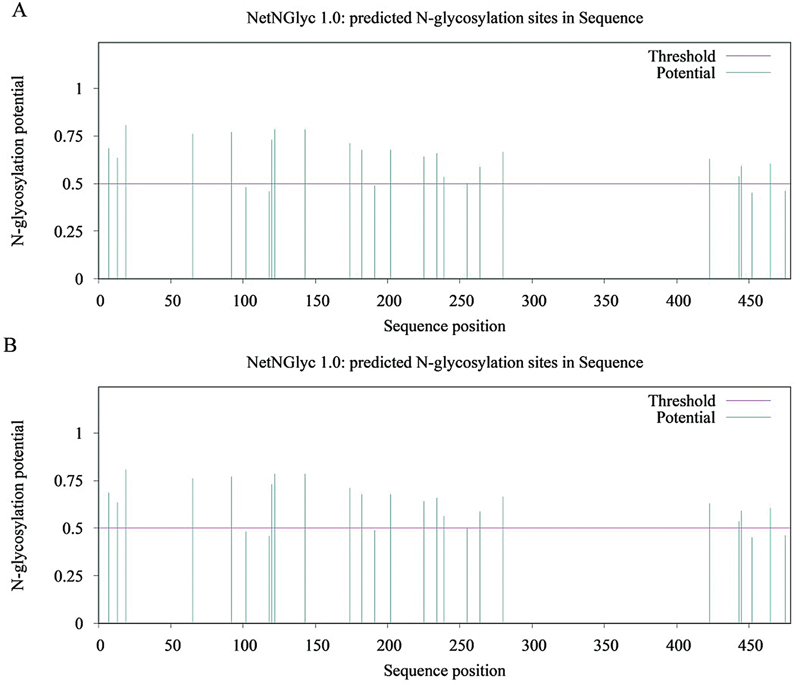
Glycosylation patterns of Trp221Arg-SERPINC1 protein: ( **A** ) Wild-type and ( **B** ) mutant type.

The analysis using PyMOL software revealed that the Threonine at position 221 of the wild-type AT protein forms three hydrogen bonds with the Alanine (Ala) at position 217, the Isoleucine (Ile) at position 218, the Asparagine (Asn) at position 224, and the Lysine (Lys) at position 225, respectively ( Fig. 3C ), while it forms two hydrogen bonds with the Asparagine (Asn) at position 224 and the Lysine (Lys) at position 225 ( Fig. 3D ). According to the guidelines of the American College of Medical Genetics and Genomics (ACMG), the c.661T > C,p.(Trp221Arg) variant was assessed as likely pathogenic (PM2_Supporting + PP1 + PP3_Moderate + PP4_Moderate). The variation has not been reported in multiple general population databases, indicating that it is a rare variant. The variation co-segregates with the related disease phenotype in the pedigree of the individual tested (mother and brother). Multiple bioinformatics prediction software tools predict that this variation is deleterious. Specifically, REVEL gives a score of 0.88, indicating that the variation is deleterious (rated as moderate). 11 The clinical phenotype, hematological indicators, and family history of the carrier of this variant are consistent with thrombophilia caused by antithrombin III deficiency related to the SERPINC1 gene. A variation involving different base substitutions at the same codon, leading to different amino acid changes (c.662G > C, p.Trp221Ser, classified as VUS), has been reported in a family with venous thrombosis. 12 And no relevant case reports involving this variation have been found in the literature or the ClinVar database. 13 The glycosylation analysis is shown in Fig. 4 . The variation did not alter the potential glycosylation sites of the protein.

## Discussion

AT is a member of the serine protease inhibitor superfamily and plays a crucial role in the physiological inhibition of coagulation proteases. Its inhibitory activity is significantly enhanced upon binding to heparin or similar glycosaminoglycans. Hereditary AT deficiency is an autosomal dominant disorder, categorized into type I and type II. 14 Type II is further subdivided into reactive site defects, heparin-binding site defects, and pleiotropic defects. Decreased AT concentration or functional loss caused by specific gene variants results in primarily presenting as pulmonary embolism and DVT. In the Chinese population, AT deficiency is the most common genetic risk factor among VTE patients, higher than PC and PS deficiency. 15 In this study, we confirmed that both the proband and her younger brother carried a compound inheritance of SERPINC1:c.661T > C,p.(Trp221Arg) heterozygous variants and PROC:c.572_574del,p.(Lys193del) variants. The proband's mother carried the SERPINC1 heterozygous variants and the father carried the PROC heterozygous variants. The proband, her brother, and mother all had a history of DVT and required long-term rivaroxaban treatment, whereas the father didn't. Phenotypically, the proband's current mutations are harmful and autosomal dominant, while the father's variant is autosomal recessive.

Decreased plasma AT activity and levels may be congenital or secondary to various conditions. 16 Clinically, liver dysfunction can reduce AT synthesis, nephrotic syndrome can lead to excessive AT loss, sepsis with disseminated intravascular coagulation can result in excessive AT consumption, and major surgery or extracorporeal interventions (such as heparin therapy) can reduce AT levels. In this study, family members ruled out acquired AT deficiency because of normal liver or kidney and no evidence of major surgery or special medication. Coagulation tests indicated the proband's mother and brother had low AT activity with normal PC and PS activities, suggesting hereditary AT deficiency, and it was confirmed by sequencing later.

The proband presented with multiple venous thromboses and significant symptoms early, and family members with single heterozygous variants exhibited related clinical symptoms, except for the father who did not have thrombosis. Gene analysis of family members revealed that the proband and her brother carried both heterozygous variants SERPINC1:c.661T > C,p.(Trp221Arg) and PROC: c.572_574del,p.(Lys193del), with AT levels reduced to 49 and 50%, respectively. The mother carried the SERPINC1:c.661T > C,p.(Trp221Arg) variant, with AT levels at 42%. The father carried the PROC:c.572_574del,p.(Lys193del) heterozygous variant, with normal AT levels. All other family members had a history of thrombosis and required long-term anticoagulant therapy. Clinical data and genetic pedigree analysis indicated that there were no underlying diseases among family members, all of whom exhibited normal coagulation parameters but synchronized decrease in AT levels. All affected members carried the same variant points, suggesting that the proband inherited both gene variants from her parents, leading to early clinical symptoms. The c.661T > C variant was identified as the primary pathogenic variant.

To further validate the impact of the c.661T > C,p.(Trp221Arg) variant on the structure of the AT protein, we carried out an extensive protein modeling analysis. This analysis did not detect any alterations in the protein's structural framework. However, it did indicate a potential reduction in stability, which is attributed to a decline in the number of hydrogen bonds. This decrease in stability could have an impact on the interaction between AT and thrombin, a key factor in the anticoagulant mechanism of heparin. It is well established that AT activity is indispensable for the anticoagulant effects of heparin, a medication commonly used to prevent and treat thrombotic disorders. A decrease in AT activity, as may be caused by the variation, could substantially attenuate the anticoagulant effects of heparin. This emphasizes the critical role of the AT protein in heparin's mechanism of action and the importance of maintaining its integrity and functionality. Further research is necessary to fully understand the implications of this variant on heparin's efficacy and to explore potential strategies to mitigate any adverse effects.

Glycosylation is one of the most common posttranslational modifications of the proteins influencing functional activity. 17 Glycosylation disturbance is a well-known pathogenic mechanism in AT deficiency. Previous studies suggested that additional glycosylation sites increase protein weight and potentially alter folding and conformation. 18 Fitches et al hypothesized that mutant proteins remain intracellular, leading to a deficiency in blood, 19 with only normal proteins entering circulation. This correlates with our clinical findings: the proband was diagnosed with type I AT deficiency due to consistently low blood AT levels. Genetic testing for rare diseases showed that hereditary AT deficiency is a monogenic disorder with at least 220 pathogenic variants in the SERPINC1 gene. Notably, the SERPINC1 gene contains one hotspot: c.881G > T(p.Arg294Leu) and c.883G > A(p.Val295Met), prevalent in Chinese VTE patients. 20 The p.Pro305His variant has also been detected in several Indian families but no hotspot variants have been identified in Caucasians. 21 Polyak et al 12 reported a different mutation at the same position, c.662G > C (p.W221S), in a proband who had been diagnosed with hereditary type I antithrombin (AT) deficiency. The primary distinction between these two mutations lies in the fact that the mutation identified in our study did not introduce an additional glycosylation site, whereas the p.W221S mutation led to the creation of an extra glycosylation site. Most gene variants described are found in individual families. This study's variant, p.Trp221Arg, impacts structure and post-translational modifications with uncertain results. The 219-221 amino acid positions contain an additional glycosylation site, possibly preventing glycosylation at the typical 224-226 site due to steric hindrance.

Protein homology analysis showed that Trp221 is highly conserved across homologous species (mammals, reptiles, birds, fish, etc.), indicating its critical role in maintaining normal AT structure and function. The proband also carried the PROC:c.572_574del,p.(Lys193del) heterozygous variant, which has a higher frequency in the general population, with not all carriers showing reduced protein C activity, possibly related to incomplete penetrance. Literature reports this variant as a risk factor for venous thrombosis and ischemic stroke, primarily in East Asians, not strictly following Mendelian inheritance. 22 23 This in-frame deletion of three bp is expected to shorten the encoded protein in the C-terminal region of the light chain which harbors a crucial functional motif essential for its interaction with protein S, as well as factors Va and VIIIa. 24 Based on clinical presentations and coagulation indicators of family members, the primary pathogenic variant is c.661T > C, as all carriers exhibit thrombosis.

In conclusion, a comprehensive phenotypic and genetic analysis was conducted on a family suffering from hereditary AT deficiency, which suggested that the proband's development of DVT could be linked to inheritance of SERPINC1:c.661T > C,p.(Trp221Arg). However, it is evident that additional in vitro experiments are required to fully uncover the precise mechanisms responsible for the decreased levels of AT in the peripheral blood.

## Notes

**What is known about this topic?**

- Inherited deficiency of the antithrombin (hereditary antithrombin deficiency, AT deficiency, OMIM #613118) is a relatively rare (1:2,000–3,000) autosomal-dominant disorder with high risk of venous thromboembolism.
- The gene that encodes for AT is known as SERPINC1 and is located on chromosome 1q23-25. This gene consists of a total of six introns and seven exons. Most of AT-deficient patients carry heterozygous SERPINC1 variants.
- Studies conducted in an experimental mouse model have demonstrated that a homozygous null variant in the SERPINC1 gene leads to embryonic lethality, attributed to bleeding complications and severe thrombosis. Over 300 genetic variants responsible for antithrombin deficiency have been discovered for the SERPINC1 gene. Variants of SERPINC1 gene lead to abnormal levels of AT in the peripheral blood.

**What does this paper add?**

- In this research, we documented the clinical presentations of the proband and her family, focusing on recurrent venous thromboembolism (VTE).
- Using whole-exome sequencing, we discovered a novel pathogenic variant in SERPINC1, which was found to co-segregate with AT deficiency in the affected family members. This discovery adds to the growing list of known SERPINC1 mutations and offers potential new directions for genetic testing and personalized management in patients with hereditary AT deficiency.
- Our study delves into both the clinical and molecular aspects of this disorder. The genetic analysis is supplemented by bioinformatics and structural modeling, which provide deeper insights into how this mutation may disrupt AT function.
